# Effects of thoracic epidural analgesia on plasma cAMP and cGMP levels in patients with heart failure

**DOI:** 10.1186/1749-8090-8-217

**Published:** 2013-11-26

**Authors:** Qing-Shu Li, Feng-Qi Liu

**Affiliations:** 1 Department of Cardiology, First Affiliated Hospital of Harbin Medical University, Harbin 150001, China

**Keywords:** Heart failure, Signal transduction, Thoracic epidural analgesia

## Abstract

**Background and aim:**

The progression of heart failure is affected by several factors, including chronic stimulation of the β-adrenoceptor. This clinical study was designed to measure the effects of thoracic epidural analgesia (TEA) on the plasma levels of norepinephrine (NE), cAMP, and cGMP in patients with heart failure and assess the clinical implication of TEA.

**Methods:**

Forty patients with heart failure were randomly assigned to TEA (TEA plus standard care) and control groups (standard care). The plasma concentrations of cAMP, cGMP, brain natriuretic peptide (BNP), and NE were measured using ELISA before treatment, the second and fourth weeks of treatment.

**Results:**

The plasma concentrations of cAMP, cGMP, BNP, and NE in the TEA group were significantly reduced by the fourth week compared to their initial concentrations (P < 0.01, for all parameters) and the control group (P < 0.05, P < 0.05, P < 0.01, and P < 0.05, respectively). The values for left ventricular end diastolic diameter (LVEDD), ejection fraction (EF), and fractional shortening (FS) in the TEA group improved significantly compared to their initial values and the control group. However, the changes in levels for these indices in the control group were no statistical significant compared to the initial levels.

**Conclusions:**

TEA can effectively decrease the plasma concentrations of cAMP and cGMP and improve cardiac function in patients with heart failure. The decreased levels of NE and cAMP occurred before the improvement in cardiac function, indicating that the abnormal epidural signal transduction can be corrected in patients with heart failure.

## Background

Heart failure is the final stage for several types of heart diseases and many related illnesses. Although the use of cardiotonic drugs, diuretics, β-blockers, and angiotensin-converting-enzyme inhibitors (ACEI) can improve the cardiac function in patients with heart failure [1-3], the morbidity and mortality of heart failure continues to remain high [4,5]. Therefore, it is necessary to search for new treatments for heart failure. In the past two decades, extensive investigations have developed several effective approaches for reducing the morbidity and mortality of patients with congestive heart failure. These investigations include pharmacological [2,3,6] and cardiac device therapies [7], but significant improvement on the clinical outcome has not been observed. Recently, cell-based therapy has been demonstrated as an effective method for treating heart failure *in vitro* and *in vivo*[8].

Sympathetic hyperactivity is a compensatory response to cardiac function that can activate several signal transduction pathways [9], but eventually becomes part of the disease process and may result in decreasing cardiac function. It was speculated that inhibiting the hyperactive sympathetic nervous system would correct the abnormal signal transduction and improve the cardiac function in patients with heart failure. High thoracic epidural analgesia (TEA) has been used extensively to treat myocardial infarction patients with severe chest pain, with the major mechanism of action being selective blockading of cardiac sympathetic [10,11]. Kock *et al.* have reported that TEA can improve the left ventricular function during stress-induced myocardial ischemia in patients with coronary artery disease [12]. In addition, we have previously reported a beneficial outcome by one patient treated with TEA [1]. Based on these preliminary results, this study was designed to demonstrate the therapeutic efficacy and safety of the TEA treatment in a clinical trial setting. The results will be useful for improving the clinical outcome of TEA in patients with heart failure.

## Methods

### Patients and study design

The design for this study and clinical protocols were reviewed and approved by the Ethics Committee of First Affiliated Hospital of Harbin Medical University, Harbin, China. All patients provided written informed consent before participating in this study.

Forty patients with heart failure that were admitted to our department between May 2010 and February 2011 were randomly divided into two groups: (1) the thoracic TEA group and (2) the control group, using a computer-generated random number. The classification was carried out by experienced physicians who were unaware of the group assignments for the patients and their laboratory test results. The patients in the control group were treated with conventional medications (e.g., digoxin, diureticum, β-blockers, and ACEI), while patients in the TEA group were treated with TEA in addition to conventional medications. Overall, medications administered to the two groups were similar, and includedβ-blocker and ACE inhibitor.

### Treatment procedure for thoracic epidural analgesia (TEA)

Blood pressure, oxygen saturation, and electrocardiogram were continuously monitored during placement of the epidural catheter. While the patients were in a sitting position, a thoracic epidural catheter was inserted at the T4-T5 interspace, and then advanced 4–5 cm cephalad until the T1-T2 interspace was reached. The catheter was connected to a bacterial filter, which was changed twice per week. Lidocaine was administered epidurally (5 ml every 2 h from 9 a.m. to 11 p.m.) to induce blockade of cardiac sympathetic segments (T1-T5), which was evaluated using cold discrimination with ice. We confirmed the catheter placement using a computed tomography scan. The epidural top ups had been provided by experienced physicians.

In addition to the standard care, patients in the TEA group were administrated 0.5% lidocaine (5 ml every 2 h from 9 a.m. to 11 p.m.) via the epidural catheter for four weeks. Patients in the control group were placed with the catheter and administrated 0.9% NaCl (5 ml every 2 h from 9 a.m. to 11 p.m.). The adequacy and level of the block were established by confirming the loss of pin prick sensation and warm/cold discrimination [13]. All the patients tolerated the treatment, no additional therapy was needed. If needed, the treatment may be administered for a period longer than four weeks. However, in this study, we only used the data for a four-week period for all the patients. The use of diuretics was decided by the physicians who had no roles in the study design or outcome evaluation.

### Plasma sampling and analysis

Blood samples were collected in a tube containing ethylene diamine tetra acetic acid (EDTA) from all the patients at admission and at the second and fourth weeks during the treatment process. Samples were immediately centrifuged at 4°C, at 3000 rpm, and the plasma was collected and stored at -80°C until being analyzed. Concentrations of plasma cAMP and cGMP were measured using an ELISA kit [Biocalvin Company, SuZhou, China] as instructed by the manufacturer. ELISA also was used to assay the plasma concentrations of norepinephrine (NE) and brain natriuretic peptide (BNP). The biochemical tests were performed by experienced laboratory assistants that were unaware of the group assignments for the patients and other laboratory test results.

### Cardiac ultrasonography

All patients underwent cardiac ultrasonography the day prior to and following treatment. The left ventricular end diastolic diameter (LVEDD), ejection fraction (LVEF), and fractional shortening (FS) were measured at these times. The ultrasonography was performed by an experienced ultrasound technician. The GE Vivid5 color ultrasonic diagnostic instrument was used, with the probe frequency 1.7-3.6 MHz. By using M-type ultrasonic, we measured the ejection fraction, fractional shortening, and left ventricular end diastolic diameter for each patient.

### Statistical analyses

Statistical analyses were performed using the statisitical package SPSS 19.0 (SPSS Inc, Chicago, IL, USA). Enumeration data were compared with the chi-square test. Measurement data were presented as mean ± standard deviation (SD) after a normality and homogeneity of variance test. A natural logarithm transform was performed on the non-normal-distributed data. Multiple group comparisons were performed using one-way ANOVA analysis of variance with Bonferroni correction. Comparisons of changes in cardiac functions before and after treatment between the TEA group and the control group were performed using a two-tailed paired Student’s t test. The statistical significance level was set to p < 0.05. A retrospective power analysis was performed for non-significant results.

## Results

The baseline characteristics for patients in each group are presented in Table 1. Forty patients (including 32 males and 8 females) with heart failure were randomly divided into two groups: (1) the thoracic TEA group (including 17 males and 3 females) and (2) the control group (including 15 males and 5 females). In the TEA group, there were five cases of coronary artery heart disease (CHD), four cases of hypertension, and 11 cases of dilated cardiomyopathy (DCM); the corresponding cases for these diseases in the control group were four, seven, and nine, respectively. The patients were classified according to the New York Heart Association as class IV (consisting of 28 patients, 13 in the control group and 15 in the TEA group) and class III (consisting of 12 patients, 7 in the control group and 5 in the TEA group). There were no statistical significance for baseline characteristics between the two groups (p > 0.05).

**Table 1:** Baseline characteristics of the patients with heart failure in the TEA group and control group

	**TEA group (n = 20)**	**Control group (n = 20)**	***p***
Median (range)	69 (79-60)	68 (78-58)	
**Gender**			
Male	17	15	>0.05
Female	3	5	
**Concomitant diseases**			
CHD	5	4	>0.05
Hypertension	4	7	
DCM	11	9	
**NYHA**			
III	5	7	>0.05
IV	15	13	

### Plasma levels of NE, cAMP, cGMP, and BNP

**Figure 1:**
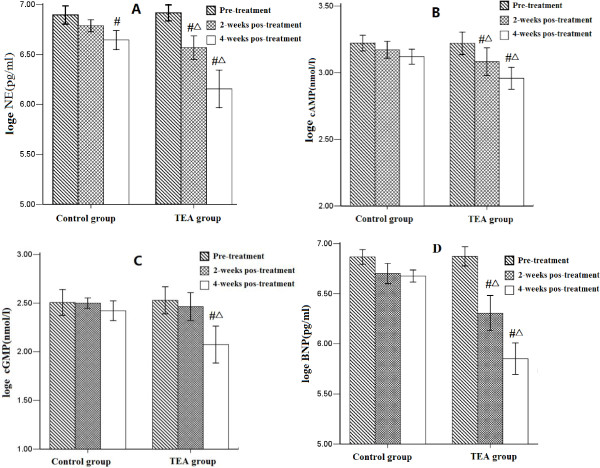
**Plasma levels of NE (A), cAMP (B), cGMP (C), and brain natriuretic peptide (D) in the TEA groups and control groups after 4-weeks treatment.** The data were transformed to a natural logarithm for a normal distribution. #: Significant change compared to pre-treatment by one-way analysis of variance with Bonferroni correction (p < 0.05). Δ: Significant change compared to control group by one-way analysis of variance with Bonferroni correction (p < 0.05).

Plasma levels of NE, cAMP, and cGMP in patients two weeks after TEA treatment was initiated were significantly lower than the pre-treatment levels (Figure 1); plasma levels at four weeks were lower than at two weeks. No significant changes were measured in the control group for these parameters following standard treatment (i.e., without TEA). Plasma levels of BNP in the TEA group decreased significantly after treatment compared to the initial levels, thus indicating that cardiac function had significantly improved in this group.

### Clinical outcome

No significant changes were observed between the two groups in the etiology of heart failure. The heart rate and blood pressure of the patients in the TEA group were decreased after the injection of lidocaine. The angina and dyspnea and other signs of heart failure in these patients gradually improved with the treatment. After four weeks of treatment, the heart function by each patient was re-evaluated. In both groups, heart function improved compared to the initial measurement but there was only a significant improvement in the TEA group.

Based on test results for each treatment group, the control group included 10 patients in class IV, six in class III, and four in class II; the TEA group included three patients in class IV, five in class III, and 12 in class II. It is worth noting there was a statistically significant difference between the two groups with respect to classification of the patients.

EF and FS increased, and the LVEDD decreased after TEA treatment (Table 2), a finding consistent with the clinical improvement of cardiac function. During treatment, two patients in the TEA group had bleeding complications, and one patient had infectious complication. No patients had venous thromboembolism complication.

**Table 2:** Changes in levels of cardiac function before and after four weeks treatment between the TEA group and control group

**GROUP**	**LVEDD (mm)**	**95% CI**	**EF (%)**	**95% CI**	**FS (%)**	**95% CI**	**NYHA**	**95% CI**
**Control group**								
Pre-treatment	64.85 ± 5.86	(53.56-76.13)	32.45 ± 7.21	(18.31-46.59)	25.80 ± 5.73	(14.56-37.04)	3.65 ± 0.48	(2.69-4.61)
post-treatment	63.15 ± 5.23	(52.89-73.40)	33.15 ± 5.98	(21.44-44.86)	26.61 ± 5.52	(15.73-37.37)	3.3 ± 0.8	(1.73-4.87)
**Chang**	1.65 ± 0.64		0.55 ± 0.38		0.85 ± 0.61		0.35 ± 0.10	
**TEA group**								
Pre-treatment	66.45 ± 5.28	(56.08-76.80)	30.35 ± 6.78	(13.23-46.78)	24.20 ± 3.54	(17.24-31.15)	3.75 ± 0.44	(2.87-4.62)
post-treatment	58.30 ± 5.38	(47.75-68.64)	34.15 ± 6.12	(16.39-50.42)	28.15 ± 4.67	(18.99-37.30)	2.55 ± 0.76	(1.06-4.04)
**Chang**	8.15 ± 0.65**		3.80 ± 0.56**		3.95 ± 0.62**		1.2 ± 0.16**	

Significant differences were performed using a two-tailed paired Student t test. (**: p<0.001, vs. control group).

## Discussion

Data that has previously been collected indicates the activation of the sympathetic nervous system, as a compensatory response to restore cardiac output, exists in the early stage of heart failure, even prior to clinical symptoms [14]. In addition, there is accumulating evidence supporting the notion that chronic activation of the sympathetic nervous system induces adverse effects on cardiac contractile function. For example, Liang *et al. *[15] reported that excessive sympathetic stimulation is associated with the development of β-receptor down-regulation and β-adrenergic sub-sensitivity in right heart failure. Patel *et al. *[16] reported that chronic infusion of NE caused myocardial hypertrophy, which reduced the inotropic response to β-receptor stimulation. Excessive levels of circulating catecholamines have been shown to cause myocardial hypertrophy, myocyte damage, and cardiomyopathy [4,17,18]. Based on these findings, excessive sympathetic stimulation induced changes in the noradrenaline-β-receptor-cAMP signal transduction axis, damaged systolic function, and induced cardio reconstruction. Therefore, blocking excessive activated sympathetic signal transduction may be an effective approach to improve cardiac contractile function.

In recent years, patients with heart failure have been treated with β-receptor blockers to block abnormal sympathetic signal transduction, which has resulted in a notable improvement in cardiac function and clinical outcome [9]. Luzza *et al*. [19] suggested the correction of neurohormonal dysfunction is a rational therapeutic approach in patients with chronic heart failure. Although there is abundant evidence that demonstrates sympathetic signal transduction is excessively stimulated in patients with heart failure [20,21], there have been few studies conducted to demonstrate the effects of the blockade of the sympathetic signal transduction for treating heart failure. In this study, abnormal sympathetic signal transduction was blocked to explore the relationships between heart failure and signal transduction. We chose the intermittent injection scheme because it has a reversible sympathetic nerve function and the timing may avoid failing to recruit sympathetic drive when needed to support cardiac function as a result of removal of sympathetic nerve. The patients’ symptoms were improved all day, also in the night without routine epidural injections. Clinically relevant laboratory test parameters in combination with clinical and heart function observations were used to measure the success of this treatment.

Results of this study demonstrated plasma concentrations of NE, cAMP, and cGMP in patients with heart failure within the TEA group were significantly lower than the pre-treatment and showed a time-dependent improvement. It is speculated that these results are involved with the decrease in production of sympathetic impulses, decreased synthesis and release of NE, inhibition of NE-β-receptor-cAMP signal transduction, and decreased synthesis and release of cAMP. As expected, cardiac function of patients with heart failure improved significantly with decreased concentrations in NE and cAMP. This finding is in agreement with observations made in several other studies [22,23] where the plasma cAMP had a negative correlation with cardiac function.

It is well known that cGMP can reduce myocardial metabolism, inotropy, and function [18,24]. The effects of cGMP are mediated by direct inhibition of L-type Ca^2+^ channels, cGMP-dependent protein kinase, and cGMP-regulated cAMP phosphodiesterases [24,25]. Therefore, reduction of cGMP decreased its negative effects to the myocardium and improved cardiac function. Down-regulation of cAMP may be attributed to the inhibition of downstream signals of NE after blocking the sympathetic nerves, which results in the reduction of negative effects on cardiac function. Furthermore, the decrease in levels of plasma NE reduced the toxic effect to the myocardium and is also associated with improvement in cardiac function. Therefore, it was concluded that the blockade of sympathetic nerves corrected the abnormal signal transduction in heart failure resulting in an improved cardiac function.

BNP, which is synthesized and secreted by ventricular myocardium, has been used as a heart failure marker when the ventricular load and ventricular wall tension are increased. In recent years, BNP has been recognized to have a significant application value in the early diagnosis and assessment of severity, prognosis, and therapeutic outcome in patients with heart failure [26-29]. In this study, levels of plasma BNP in the TEA group decreased markedly after TEA treatment compared to the initial levels, indicating that cardiac function was significantly improved. This finding was consistent with other biochemical parameters and clinical heart function analyses.

TEA could lead to complications like hypotension, respiratory depression, bleeding or hematomas, infectious or abscesses, venous thromboembolism, paraplegia, and even cardiac arrest. The most common complication was hypotension, and the blood pressure patients had improved after infusion. In this study, only one patient in the TEA group had the complication of bleeding, which was improved after the compression; no patients withdrew from the study because of complications.

## Conclusion

TEA may decrease the levels of plasma NE, cAMP, and cGMP, the first messenger and second messenger of signal transduction, block the over-activated sympathetic signal transduction, and improve cardiac systolic function. It is inferred that correcting abnormal signal transduction through blocking the sympathetic nervous system is a potential treatment for patients with heart failure.

## Competing interests

The authors declare that they have no competing interests.

## Authors’ contributions

QL participated in the design of the study and performed the statistical analysis, carried out the immunoassays, and drafted the manuscript. FL conceived of the study, and participated in its design and coordination and helped to draft the manuscript. All authors read and approved the final manuscript.
